# Social Capital Theory, Social Exchange Theory, Social Cognitive Theory, Financial Literacy, and the Role of Knowledge Sharing as a Moderator in Enhancing Financial Well-Being: From Bibliometric Analysis to a Conceptual Framework Model

**DOI:** 10.3389/fpsyg.2021.664638

**Published:** 2021-05-21

**Authors:** Asha Thomas, Vikas Gupta

**Affiliations:** Delhi School of Management, Delhi Technological University, Rohini, India

**Keywords:** financial well-being, social capital theory, social cognitive theory, social exchange theory, bibliometric (R-package)

## Abstract

A person’s financial well-being (FWB) is the complete contentment gained from one’s present financial condition. This has a powerful impact on the entire achievement of an employee’s “well-being.” Researchers, financial analysts, financial planners, educationists, and economists have explored the “enablers” to improve employees’ living standards by investigating the possible “FWB” resources for decades. There is no literature available to show the connection between social capital theory, social exchange theory (SET), social cognitive theory (SCT), financial literacy and FWB, and employees’ financial knowledge sharing a moderator to expand the complete FWB.

## Introduction

Financial well-being (FWB) has recently become a hot topic (Joo and Grable, 2004; Shim et al., 2009; Brüggen et al., 2017; Netemeyer et al., 2018; Collins and Urban, 2020). The first quarter of 2020 was severally impacted by the COVID-19 pandemic that influenced the domestic, financial, and commercial lives of the people (Barrafrem et al., 2020; Sharma et al., 2020; Botha et al., 2021). Policymakers placed financial resilience and FWB on the top of the schedule. This offered a possibility to re-concentrate on the important factors of “financial literacy” (OECD, 2020). With savings rates at record lows and inadequate long-term financial planning for retirement (Taft et al., 2013), FWB has become an important topic for individuals and households as well as for societies and countries. Research on the topic, however, remains scarce and scattered across disciplines (Brüggen et al., 2017; Mahendru et al., 2020). There are very few researches on the aspects relating to how FWB aids an organization’s employees (Choi et al., 2020; Sabri et al., 2020). FWB means a person’s traits, monetary attitude, and monetary “stressor” occasion. Financial actions result from FWB (Kim et al., 2003). Today, the role of employees in handling their finances is complicated. Thus, there is a disparity in FWB between the employees’, and the same can negatively impact an organization’s competitiveness (Sabri et al., 2020). Employees’ who are not financially sufficient are always tensed and distracted. This affects the absence, competence, retirement, and medicinal expenditures of an employee. Therefore, the main objective of this study is to focus on employees’ FWB determinants.

The present research helps understand the important aspects that can aid employees’ FWB in an organization or workplace. There is a need for improving ways of “financial management,” particularly for the working employees because of the augmented levels of the present individual “indebtedness,” and focus more on an individual’s obligation for “financial planning” between them (Lusardi and Olivia, 2011). This research aids in combining numerous pieces of literature relating to the subject and emphasizing the critical resources and records. Therefore, it is vital to bring forward significant predictors to help employees have assurance in the job and promote their FWB. Terms like “financial wellness,” “financial satisfaction,” etc. are used in this present study. It is significant to state that “FWB” is often substituted with terms like “financial wellness,” “financial satisfaction,” etc., in the literature (Mahendru, 2020). In this research, we have included these terms as wider concepts and for literature.

The present study carries out the bibliometric analysis of literature studies relating to FWB. The present research talks about the following questions regarding the research conducted on the printed material on FWB: (1) What is the pattern of annual publication trends? (2) What is the pattern of collaboration and co-citation trends? (3) Which are the most productive countries? (4) Which are the most trending scientific journals? (5) Which are the most frequently used author keywords and themes in the literature? The responses to these questions aid in directing the present study. The author has gone a step ahead and has combined a conceptual model to help future researchers better. This will further facilitate a better understanding of the FWB.

The present study has created a conceptual model that covers significant predictors that allow employees’ FWB with the aid of significant theories like social capital. These theories were widely talked about in sectors like knowledge sharing (KS), health, well-being, etc. (Gupta and Thomas, 2019; Ko, 2019; Berraies et al., 2020; Thomas et al., 2020). Thus, they can act as very important determinants in the FWB of the employees in the organization.

The following is the paper’s arrangement: section “Research Methodology” talks about the research methodology, and section “Bibliometric Analysis” introduces the bibliometric analysis of the active literature by recognizing the main aspects that impact the employees’ FWB in the organization by suggesting a theoretical model derived from social theories for forthcoming studies. Significant theories are examined in the theoretical underpinning in section “Theoretical Underpinning and Literature Review.” Section “Predictors and Moderators of Financial Well-being” familiarizes the vital determinants and a moderator of FWB. Section “Conceptual Model” suggests a conceptual model framework and, finally, the conclusion is presented in section “Conclusion.”

## Research Methodology

### Procedure

**FIGURE 1:**
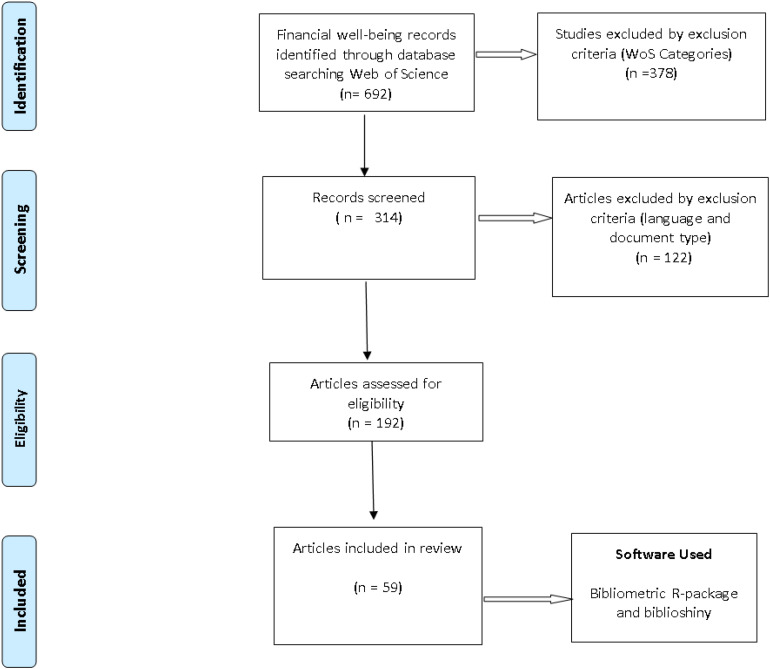
Preferred reporting items for bibliometric review framework (summary of the selection process).

To carry out a bibliometric review of the published FWB articles, data were collected from the web of science (WoS). The present research used the preferred reporting items for systematic review (PRISMA) standard (Figure 1). Keywords like “FWB,” “financial satisfaction,” and “financial wellness” were used and 692 articles were selected. Employing terms like “management, business sociology, economics, family studies, psychology, psychology clinical,” in English and document type (articles, early access, and review) presented with 314 articles. After reviewing the title and the abstract of the paper, only 192 researches were chosen for the study.

This research incorporated bibliometric analysis on 192 scientific research papers that were culled out from the online WoS database relating to the theme “FWB;” bibliometric R-package and biblioshiny were applied to attain and document the quantitative data relating to the numerous articles chosen; and based on a detailed review, this study proposes a conceptual model and testable propositions.

Bibliometric methods apply “bibliographic data” from databases available online. The bibliometric analysis is based on data that permits scientific research and presents a complete vision of scientific research. The data relating to the bibliography is readily available and has augmented bibliometric reviews in distinct studies. This method has been applied to numerous researches. Many software tools are available and are used by scientometrics to examine the bibliometric data. The bibliometrics select a tool depending on the kind of analysis needed. Bibliometrix can be applied for both examining and planning bibliographic data. Knowledge workers can evaluate, alter, and enhance Bibliometrix as it is an “open-source software” written in R-packages, a prominent society of developers and users, and presently has over 16,000 software packages. Bibliometrix can, thus, be applied as part of a superior and broad data analysis workflow. The necessary details of extracted documents by WoS for bibliometric analysis are shown in Table 1.

**TABLE 1:** Key details of extracted documents by WoS for bibliometric analysis.

Description	Results
**Main information about data**	
Timespan	2001–2021
Sources (Journals, Books, etc.)	115
Documents	192
Average years from publication	4.94
Average citations per documents	12.73
Average citations per year per document	1.979
References	8577
**Document types**	
Article	171
Article; early access	16
Article; proceedings paper	1
Review	2
Review; early access	2
Document contents	
Keywords plus (ID)	613
Author’s keywords (DE)	571
**Authors**	
Authors	556
Author appearances	599
Authors of single-authored documents	29
Authors of multi-authored documents	527
**Authors collaboration**	
Single-authored documents	32
Documents per author	0.345
Authors per document	2.9
Co-authors per document	3.12
Collaboration index	3.29

*Bibliometrix (R package).*

## Bibliometric Analysis

### Average Article Citations Per Year

**FIGURE 2:**
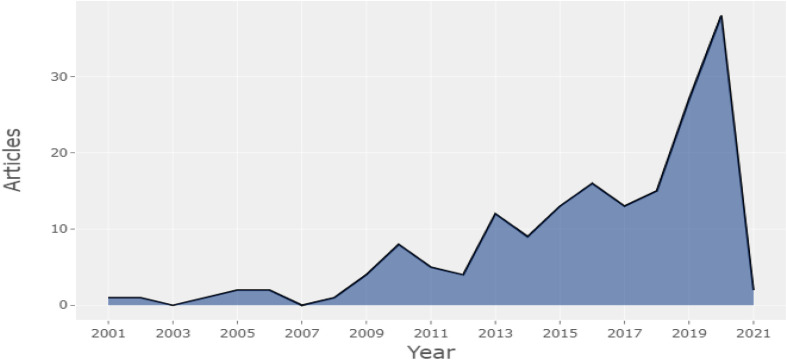
Average article citations per year.

Information on 192 articles that were published between 2001 and 2021 was culled out from the WoS database. The “analysis period” spreads over 20 years of scientific production, but the actual enhancement in published articles happened in the last 4 years (Figure 2). The annual growth rate was 3.93% with 27 publications in 2019 and 38 publications in 2020.

### Word Dynamic

**FIGURE 3:**
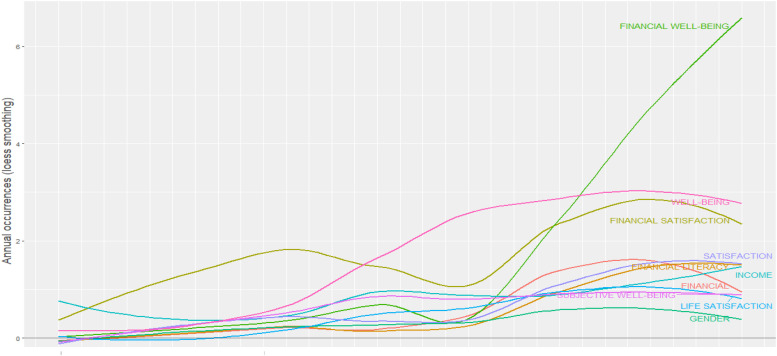
Word dynamic graph.

The authors examined the occurrence of the keywords “time-dependent occurrence.” Figure 3 shows that the amount of “main-term occurrences per year” increased eventually, but several expanded vigorously compared to others. The terms with the top happenings were “FWB,” “well-being,” and “financial satisfaction.”

### Relations Between Author Keywords, Authors, and Source

**FIGURE 4:**
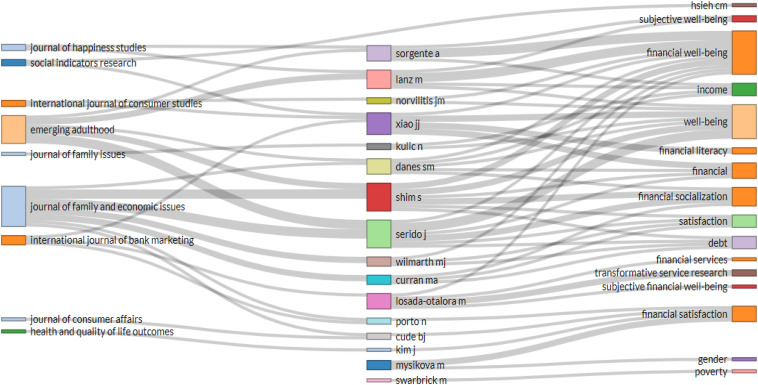
Three-field plot of relations between author keywords, authors, and source.

Figure 4 depicts the illustration for researching in the literature relating to FWB and focuses on associations between the primary keywords used by authors, creators, and origin. It is shown by the analysis where the authors had often published, studied, and investigated the FWB concepts. Topics relating to research were considered as keywords. Examination of top authors, origins, and keywords showed three authors, namely, Xiao and O’Neill (2018); Shim et al. (2009); and Serido et al. (2010); and two sources, namely, *Emerging Adulthood* and *Journal of Family and Economic Issues* have a strong association with the literature relating to FWB’s primary research themes, particularly “FWB,” “well-being,” “financial socialization,” and “financial satisfaction.”

### Co-citations and Most Relevant Sources

**FIGURE 5:**
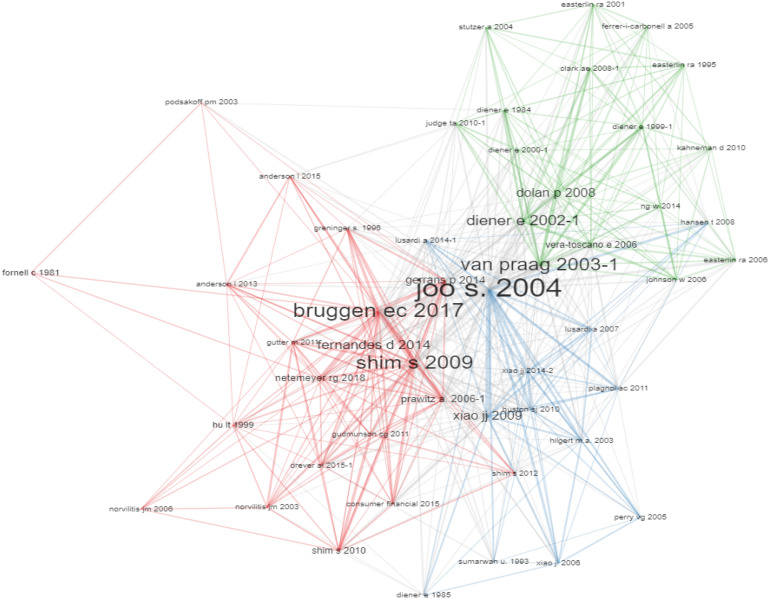
Co-citation network.

The organization of the co-citation network depicts that Joo and Grable (2004), Brüggen et al. (2017), and Shim et al. (2009) are the most referred to people. The organization of the network comprises groups in which each color is a “component” (Figure 5).

Table 2 presents the top 20 journals that are most cited and suggestive of the journal quality in this area. The most influential sources relating to *Social Indicators Research* and *Journal of Family and Economic Issues* are presented.

**TABLE 2:** List of top 20 journals.

Sources	Articles
*Social Indicators Research*	13
*Journal of Family and Economic Issues*	12
*International Journal of Bank Marketing*	9
*Journal of Consumer Affairs*	7
*Emerging Adulthood*	6
*Journal of Economic Psychology*	6
*International Journal of Consumer Studies*	4
*Journal of Happiness Studies*	4
*Journal of Family Issues*	3
*Journal of Service Research*	3
*Acta Sociologica*	2
*Applied Economics Letters*	2
*Children and Youth Services Review*	2
*Ekonomicky Casopis*	2
*European Journal of Finance*	2
*Frontiers in Psychology*	2
*Health and Quality of life Outcomes*	2
*Health Economics*	2
*International Journal of Aging and Human Development*	2
*Journal of Behavioral and Experimental Economics*	2

### Thematic Map

**FIGURE 6:**
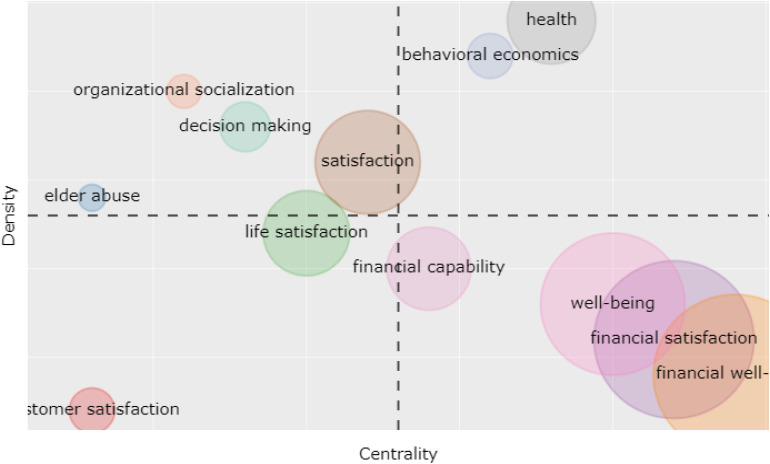
Thematic map.

The typological themes are presented on a two-dimensional plot in the thematic map (Cobo et al., 2011). Keywords are generated in this research on the basis of co-word analysis. These themes can be categorized into four quadrants on the two-dimensional graph with aspects—centrality and density. Bubble on the map presents each theme. “Behavior economics,” “health,” “financial literacy,” “wealth,” and “information” are presented in bubbles on the graph (Figure 6). The motor theme relates to “behavioral economics” and “health,” and is presented in the upper-right quadrant with high density and centrality and the same is at the center of the regulation. It is the most extensively “talked about” topic. The central theme in the lower-right quadrant includes “financial capability,” “well-being,” “financial satisfaction,” and “FWB.” This is a significant but not “well-developed” section. The upper-left quadrant shows three functional themes, i.e., “organizational socialization,” “decision-making,” and “satisfaction,” and these are well-developed and linked internally but have low external bonds and are unimportant. Themes relating to the contentment of life and customer’s contentment are presented in the lower-left quadrant and the same are weak and insignificant. It depicts both the rising and waning bonds.

### Country Total of Articles

**FIGURE 7:**
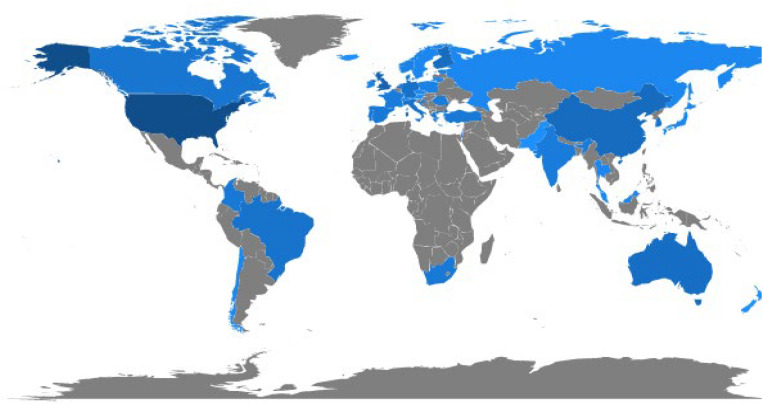
Country analysis.

Figure 7 shows the countries that contemplate the FWB theme. The maximum articles on the FWB are from the United States of America, followed by the United Kingdom, China, Finland, Germany, the Netherlands, Italy, and Brazil. This theme has expanded in countries that are situated on distinct continents and emphasize a rising interest in FWB. In Figure 7, the dispersed blue color shows an amazing growth in the FWB in various countries. Many countries like Argentina, Mexico, Iran, Indonesia, etc., are still not connected to the “scientific debate” of FWB.

### Historiography

**FIGURE 8:**
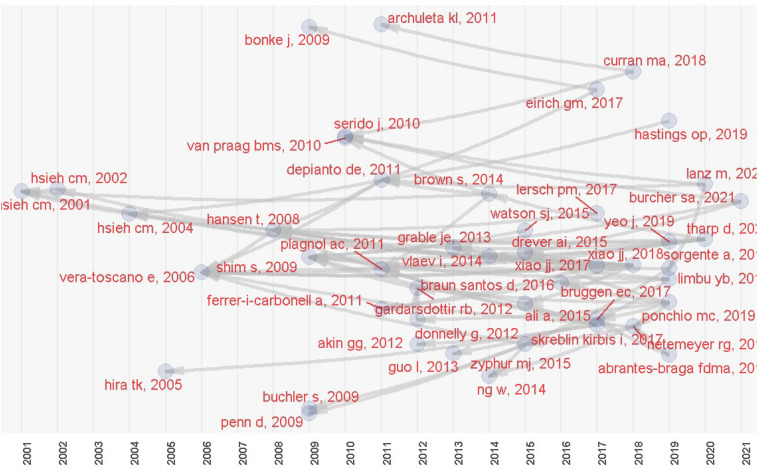
Historic direct citation network.

The historiographic analysis was conducted based on the downloaded data, and a chronological map was generated from the most pertinent citations (Figure 8). The first articles were mentioned by Hsieh (2004), Vera-Toscano et al. (2006), Hansen et al. (2008), and Shim et al. (2009).

### Most Productive Countries and Total Citations Per Country

Table 3 presents a single country publication (SCP) that provides articles that are published by the same country researchers, whereas multiple country publication (MCP) covers articles that are published by the association of other researchers who belong to distinct countries. In view of other corresponding author countries, it is observed that the top two countries are the United States with 79 articles (SCP: 69, MCP: 10) and the United Kingdom with 16 articles (SCP: 12 and MCP: 4). In countries like Turkey, Thailand, United Arab Emirates, Japan, and Malaysia, the MCP rate is zero, which shows that their study was based on the same country. Romania has the highest MCP ratio (MCP ratio = 0.667). These outcomes show that countries with a high MCP rate are receptive to international associations in research in comparison to other countries.

**TABLE 3:** Most productive countries and total citations per country.

Country	Total citations	Average article citations	Country	Articles	Freq	SCP	MCP	MCP_Ratio
United States	1308	16.56	United States	79	0.41361	69	10	0.127
United Kingdom	225	14.06	United Kingdom	16	0.08377	12	4	0.25
Finland	130	43.33	United Arab Emirates	1	0.00524	1	0	0
Iceland	86	86.00	Turkey	2	0.01047	2	0	0
Australia	63	7.88	Thailand	1	0.00524	1	0	0
China	62	7.75	Switzerland	2	0.01047	1	1	0.5
Netherlands	62	20.67	Sweden	1	0.00524	0	1	1
Spain	61	20.33	Spain	3	0.01571	2	1	0.333
Singapore	60	20.00	South Africa	5	0.02618	5	0	0
Denmark	57	19.00	Singapore	3	0.01571	0	3	1
Norway	51	25.50	Romania	3	0.01571	1	2	0.667
Malaysia	30	15.00	Portugal	1	0.00524	0	1	1
Germany	25	5.00	Poland	1	0.00524	0	1	1
Canada	22	4.40	Norway	2	0.01047	2	0	0
Italy	19	2.11	Netherlands	3	0.01571	3	0	0
South Africa	18	3.60	Malaysia	2	0.01047	2	0	0
Portugal	14	14.00	Lithuania	1	0.00524	0	1	1
Brazil	13	4.33	Korea	3	0.01571	2	1	0.333
India	13	4.33	Japan	1	0.00524	1	0	0
Croatia	11	5.50	Italy	9	0.04712	5	4	0.444

### Conceptual Structure

**FIGURE 9:**
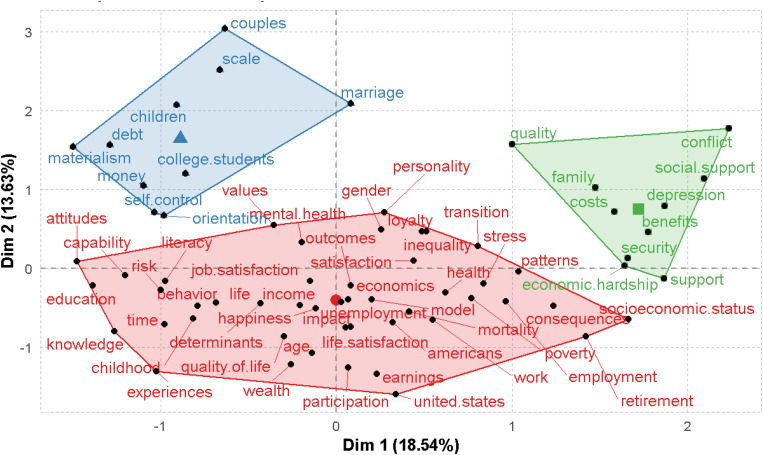
Conceptual structure (method: multiple correspondence analysis).

The bibliometrix R-package uses the conceptual structure function to conduct multiple correspondence analysis (MCA) for drawing a conceptual structure of the field and K-means clustering to recognize a bunch of documents that convey general ideas. MCA is an exploratory multivariate technique for the graphical and numerical analysis of multivariate categorical data. Homogeneity analysis of an indicator matrix is conducted by MCA to get a low-dimensional Euclidean representation of the earlier data. The words are marked on a two-dimensional map. Figure 9 shows the keywords plus conceptual structure that are connected to the FWB publications. This figure depicts that the analysis publications are grouped into three primary clusters, which show the intellectual structure of FWB literature. The results are explained on the basis of the comparative placement of the points, and these are allocated along the dimensions. Since the words are “more similar” in allocation, they are closely represented on the map.

## Theoretical Underpinning and Literature Review

The author has gone a step ahead and introduced a conceptual model. The present study has created a conceptual model covering significant predictors that allow employees’ FWB to aid significant theories like social capital, social exchange theory (SET), and social cognitive theory (SCT). Thus, understanding theories and their constructs can aid as critical factors in fostering the FWB of employees in the organization.

### Social Capital Theory

According to the social capital theory, the social association between individuals is “productive resources.” Social capital depicts the association between individuals residing in a specific society. Social capital is described as “the sum of the actual and potential resources embedded within, available through, and derived from the network of relationships possessed by an individual or a social unit” (Nahapiet and Ghoshal, 1998; Berraies et al., 2020). Furthermore, Nahapiet and Ghoshal (1998) and Tsai and Ghoshal (1998) have observed and validated the fact that social capital aids in the exchange of resources and brings in novelty in an organization. As per Nahapiet and Ghoshal (1998), there are three distinct dimensions, viz., structural social capital, relational social capital, and cognitive social capital. Structural social capital is the complete sample of association among actors (Nahapiet and Ghoshal, 1998). As stated, structural social capital covers communication that can be employed “to obtain information or access specific resources.” Relational social capital talks about the emotions and the significant aspects of the association among actors. Nahapiet and Ghoshal (1998) emphasize that joint attempts can be inspired by trust in an end and the “other actors’ support for achieving goals.” This applies to the extent where trust did not exist (p. 465). Cognitive social capital is the joint depiction, understanding, and significance among actors. Social capital was significant in the association between FWB and the contentment of life between older people (Yeo and Lee, 2019).

From Table 4, it can be said that social capital theory is mostly applied for financial wellness, health, and employee well-being (Berraies et al., 2020). Thus, this research will include “social network” and “trust” as significant factor of FWB.

**TABLE 4:** Social capital structure used in a similar context.

Literature	Structural dimension	Relational dimension	Cognitive dimension	Context
Jiménez-Solomon et al. (2016)	Social network			Financial wellness
Mithen et al. (2015)	Informal network, formal network, social support			Health
Yeo and Lee (2019)	Social ties	Reciprocal exchanges		Financial well-being
Agarwal et al. (2018)	Social interaction			Financial well-being/financial trouble
Helliwell (2006)		Social trust		Well-being
Ko (2019)	Social interaction	Trust	Shared vision	Employee well-being
Present study	Social network	Trust		Financial-well-being

### Social Exchange Theory

Social exchange theory is one of the most significant theoretical concepts to understand people’s reactions. There are sequences of communications that create responsibilities in social exchange. These communications are inter-reliant and dependent on another person’s action (Cropanzano and Mitchell, 2005). Such conversations may cover swapping actions, concrete or insubstantial reserves, as well as finances and socioeconomic results. As per Blau (1964), SET helps accept organizations’ and managers’ roles in generating employee responsibilities and optimistic work outlooks. Social exchange is related to an indefinite conversation or positive act started by an organization and is employed to treat its employees and expects that the same will be returned (Gould-Williams and Davies, 2005). Thus, an employee’s responsibility is to reciprocate by accepting an optimistic approach in the place of the favorable work environment and attractive “financial and non-financial” advantages; also, the employee has to change their negative approach at work because of negative actions (Parzefall and Salin, 2010).

Positive social exchange can provide joint advantages to the organization and employees (Ko and Hur, 2013). To comprehend “workplace behavior,” it is best to understand SET (Cropanzano and Mitchell, 2005). SET assumes that individuals get what is required and desired by exchanging with other people to reduce prices. Faithful, trustworthy, and joint associations develop eventually as per the SET principle. This is possible when particular “rules of exchange” are followed by parties. These exchange rules are a “normative definition of the situation that forms among or is adopted by the participants in an exchange relation” (Emerson, 1976). Thus, rules and exchange criteria are strategies for the process of exchange. Therefore, the SET is employed in the organizational behavior model based on the “exchange rule or principle” that the researcher relied on. The focus of the majority of management research is on “expectations of reciprocity.” The SET specifies that a particular antecedent leads to interpersonal associations at work, and these are the “social exchange relationships” (Cropanzano et al., 2001; Cropanzano and Mitchell, 2005). When employers show concern for employees, social exchange associations multiply, which, thus, produces useful outcomes. There is a constant practice of social exchange between a person and the organization. This is considered during the communication between employee and employer. Justice and understanding in FWB reward made by the employer of this swap will depict if employees are connected, whether they will continue, and whether the employees are inspired in their inner observation of justice (like advantages, job safety, association, labor substance, vocation advancement, compensation, and monetary prize) (Veldsman and Pauw, 2018).

### Social Cognitive Theory

The source of SCT is the health sciences. It presents a “human agency model” where people practically “self-reflect, self-regulate, and self-organize” (Bandura, 1989). Martin et al. (2014) believe that “SCT estimates the ability of an individual to engage in a targeted behavior, based on internal and external parameters and their interrelationships” (p. 2). The center of this theory is Triadic Reciprocity, the co-interrelation of an individual, ecological, and attitude aspects. It works as a controller and affects the attitude of an individual (Bandura, 1986). This theory suggests that ethical and other psycho-social aspects like the condition of work, weather, etc., show how ethical behavior is ruled by ethical analysis (Wood and Bandura, 1989). Thus, “SCT adopts an interactionist perspective to moral phenomena” and proposes an arrangement in which “personal factors, such as moral thought and affective self-reactions, moral conduct, and environmental factors all operate as interacting determinants that influence each other in determining outcomes” (Bandura, 1986).

### Financial Well-Being

Financial well-being is studied extensively in economics, psychology, and services marketing, yet the description or dimensions of this notion are not accepted globally in the literature (Brüggen et al., 2017). FWB has been defined by Brüggen et al. (2017) as “the perception of being able to sustain current and anticipated desired living standard and financial freedom.” The definition is unfair as it covers the level of feeling of a person’s contentment relating to monetary circumstances (Shim et al., 2009).

These processes can be calculated impartially as presented with the help of the profits, assets worth, etc. (Gatina, 2016). Yet, it is shown continuously that people with the same FWB aim may possess varied insights (Brown and Gray, 2016; Ianole-Calin et al., 2020).

## Predictors and Moderators of FWB

Significant facilitators and a moderator relating to social capital theory, SET, SCT, financial literacy, and KS derived from the literature review are presented in this section. It also gains testable proposals for upcoming studies.

### Reciprocity

Reciprocity is an essential characteristic of social exchange and social life. Reciprocity includes the giving back of advantages to another person for-profits attained (Molm, 2010). Generally, SET is extracted to understand how reciprocity promotes employees’ FWB (Blau, 1964). As per SET, when employees see that an organization encourages them, they are responsible for reciprocating (Blau, 1964; Cropanzano and Mitchell, 2005). The service connection is the swapping of reserves, reward, price, and results. This structure is employed for accepting the employee–employer relationship. Employment is a reserve and the security in work is a prize in return for employees’ investment. As per Choi et al. (2020), FWB is one of the most significant devices employed for clarifying the connection between job insecurity and monetary pressure and disclosed that FWB partially mediates the association between job insecurity and financial stress. There is a joint responsibility between two participants, the “employee and the organization,” where the employee’s reciprocal anticipation relates to responsibilities (what will the employee do for the organization?) and their rights (what does the employee want “in return?”) (Parks and Komorita, 1998). Reciprocity is a social standard (Gouldner, 1960). It affects the behavior of an individual, but everyone does not stick to this standard at the same level. Individuals respond when they think that it is correct and suitable to do so. According to Eisenberger et al. (1987), a few people will support this “reciprocity” standard in comparison to others. Individuals who endorse this standard believe in “exchange ideology or positive reciprocity beliefs.” Positive reciprocity beliefs impact three social constructs. These are justice, organizational support, and organizational identification. Employees, who believe in “positive reciprocity beliefs,” payback in the form of optimistic acts. Reciprocity, according to employees, is employed for evaluating savings and advantages. The employee looks forward to obtaining particular incentives from the organization. Similarly, the organization anticipates long-term loyalty and output from the employee (Piccoli and De Witte, 2015). When an attempt is made at the job, the same is used as part of a substituting procedure, and an organization contributes to this in terms of reward. There are three ways by which incentives are shared. These are cash, admiration, and job openings comprising job safety. Because of this, job safety is a prize for the investment made by employees’. It can be theorized that FWB is impacted by reciprocity. It is more likely that people will give to an organization believing that nothing will be lost by doing the same. This will then help in profits, remuneration, and job safety. This will further add to the employee’s FWB. Thus, it can be suggested that:

*P1: Reciprocity is connected with FWB in a positive and significant manner.*

### Social Network

Social capital is a defensive source for the addition to social networks (Gupta and Thomas, 2019; Thomas and Paul, 2019; Thomas et al., 2020). This can be influenced to preserve happiness once duplicate sources are reduced (Frank et al., 2014). Structural social capital is “an individual’s connection to, or isolation from, social networks; their level of social support in times of crisis; and their need for and access to community and/or government supports.” The impact of powerful and feeble ties in a network is associated with social capital. These social networks and ties can improve the “family” and society habitat for the people and help them to work efficiently (Fukuyama, 2001). These ties and networks can be of numerous types like “emotional support, functional support, assistance, a sense of belonging/security, job opportunity, trust, or shared values and ideas” (Coleman, 1988; Litwin, 1995; Hao and Johnson, 2000). When kids, relations, and friends provide a high level of emotional support, and there are higher levels of networks or financial assets in a society, these traits help in achieving a superior level of social stock impacting the standard of life of a person (Coleman, 1988). The stock-market contribution is affected by the “social structural” aspect as researched by Hong et al. (2004). A simple model was prepared by them, and it was found that when a “social” investor saves in the stock market, there is a higher rate of involvement among his colleagues. This model forecasts higher involvement between “social investors” in comparison to “non-social investors.” There is also an option of numerous “social equilibria.” As per Wang and Xiao (2009), people with superior social help earned less liability. Thus, the social network has a significant influence on a person’s FWB. Thus, the authors propose as follows:

*P2: Social network is connected with FWB in a positive and significant manner.*

### Social Trust

Sometimes, social trust is seen as an outcome and a catalyst of networks that are used recurrently (Torche and Valenzuela, 2011). Vital support can be offered by acquaintances, relations, and society when there exists financial stress, particularly in times like COVID-19. This can help meet essential requirements and expenditures like earnings, meet unforeseen expenses like urgent monetary help, and others like employers assisting the youth to make savings with numerous schemes. Where the social network trust is not relied upon by a few people during economic pressure, there is a low FWB. Social capital is helped with the resources and sustenance that are provided by the governments, trade, and social services and has the critical impact that helps in allowing or disallowing FWB (Orthner et al., 2004). This is a vital resource to lessen unenthusiastic incidents, maintaining firmness, recovering adaptiveness, managing abilities, and maintaining economic flexibility. A higher level of FWB is reported by people who believed and trusted other people for financial support during economic pressure. A high level of trust among the employees will foster the FWB of employees. Thus, the authors propose as follows:

*P3: Social trust is connected with FWB in a positive and significant manner.*

### Financial Self-Efficacy

As per the SCT, in a social-learning context, self-efficacy connects persons and the surroundings, and the communication between them (Bandura, 1997). It explains a person’s capability to achieve a task victoriously and anticipation of future results. Self-efficacy is a significant concept in social psychology and denotes an emotion of efficiently tackling a condition (Bandura, 1977). When the level of “self-efficacy” is high, it creates advantages for people’s happiness, primarily bodily and psychological well-being, impacting peoples’ attitude variations (Bandura, 1977, 1982). In the economic area, monetary self-efficiency is functional when monetary administration references are added into the “self-efficacy” term. In financial decision-making, self-efficacy displays a person’s faith in achieving actions needed to attain their financial purposes (Snyder and Lopez, 2009). Future objectives can include retirement or clearing bills the subsequent month (Lown, 2011). In household finance, self-efficacy connects with optimistic financial behaviors and results (Asebedo and Seay, 2018; Farrell et al., 2016). According to Farrell et al. (2016), the connection of financial self-efficacy on women’s FWB after applying an econometric model elucidates its work toward the individual’s “financial behavior.” Previous research shows an optimistic connection between financial efficacy and FWB (Shim et al., 2009). Higher levels of self-efficacy associate with FWB and set demanding objectives along with making the financial assistance easy (Bandura, 1997; Lim et al., 2014). Latest studies have depicted the significance of financial self-efficacy for forecasting more savings, “lower debt and adjustment to retirement.” Financial self-efficacy shows a “domain-specific” faith that a person is capable of making efficient monetary choices. As per Lim et al., 2014, “financial self-efficacy” may serve as significant monetary wellness plans in the campus. The outcome is that by adding to monetary “self-efficacy” efficiently, it enhances students’ monetary situation as it means taking aid for finances. The more specific a person is of one’s monetary capability, there are more promising results (Brüggen et al., 2017). Thus, financial self-efficacy is a reason to attain long-term FWB (Chen et al., 2001). Thus, the authors propose as follows:

*P4: Financial self-efficacy is connected with FWB in a positive and significant manner.*

### Financial Literacy

Generally, financial literacy involves providing people with the information and cognitive talents required to understand the economic division and manage their monetary matters (Atkinson and Flore-Anne, 2011). Psychological and attitudinal attributes help an individual to manage their finances. There is a requirement for a person to drive to look for knowledge relating to finances, the skill to manage sentiments that can impact their “decision-making” and assert their decision-making, and the ability to supervise finances. If an individual holds these characteristics, then the person will experience a positive influence over their finances in the future. He will have the momentum and the ability to act knowledgeably and sensibly to attain positive results (Farrell et al., 2016). Financial education aims to train people to administer their finances, achieve their economic goals, and shun anxiety connected to monetary issues, thereby making their FWB better. Financial education policy is a central part of the economic empowerment and people’s flexibility and adds to the economical arrangement’s complete constancy (OECD, 2020). As per the OECD/INFE definition of “financial literacy,” if a person has enough information and can act in a “financially prudent way,” their behavior will impact their choice to act. Thus, it is a combination of awareness, knowledge, skills, attitudes, and behavior necessary to make sound financial decisions and ultimately achieve individual FWB (OECD, 2020). There may be an association between FWB and financial knowledge as suggested by researches. This is interesting as this association may not always be optimistic. According to Mugenda et al. (1990), there may be a negative association between information and financial position awareness. According to them, well-informed people assess the occasion and circumstances in a very different manner in comparison to other people. This is generally depicted as the “negative along with positive aspects of the household’s financial status” (p. 355). Mugenda et al. (1990) further stated that well-informed people would attempt to augment their living standards by financial aids as they are less convinced, whereas people with less information may not understand that they are financially weak. A positive association has been highlighted by many researchers connecting financial literacy and FWB. Financial literacy and FWB have been forecasted by “academic ability” in many researches (Shim et al., 2009; Sabri et al., 2010). In a cross-sectional survey, Adam et al. (2017) used “strategy” on 400 respondents randomly from 1,500 association members. The results show that retirees’ FWB is affected by “financial literacy.” According to Lusardi et al. (2010), many young people do not have the primary “financial knowledge” required to make good choices in finances. Joo and Grable (2004) show that “financial literacy” directly impacted the FWB. O’Neill et al. (2000) also showed that if consumers are educated in primary individual finances, they may administer their finances well, which will result in better FWB. Thus, it is proposed as follows:

*P5: Financial literacy is connected with FWB in a positive and significant manner.*

### Knowledge Sharing

Knowledge sharing means when a persons’ attitude relates to imparting their information to other people (Gupta and Jain, 2017; Chopra and Gupta, 2019; Berraies et al., 2020; Thomas and Gupta, 2021). According to Jackson et al. (2006), KS is a collection of actions that convey information to other people. As per Al-Husseini and Elbeltagi (2018), “it is a process integrating two dimensions, namely, the collecting and the donating of tacit and explicit knowledge.” “Knowledge donating” is the procedure through which employees’ impart their information to other individuals (Al-Husseini and Elbeltagi, 2018; Gupta and Thomas, 2019). Knowledge collecting is the procedure in which people want and attract more knowledge from other individuals (Al-Husseini and Elbeltagi, 2018). According to Allameh (2018) “knowledge is greatly individualistic and is entrenched in specific social contexts.” Information and alertness are significant precursors for help (Lim et al., 2014). Sharing knowledge is important for researchers and practitioners. Due to its significance, numerous organizations are putting their sources, time, and finances in managing knowledge systems to endorse KS among their employees’ (Thomas and Chopra, 2020). Many researchers emphasize the aspects that persuade employees’ to impart information. Along with these factors, employees’ well-being is essential to increase KS in organizations (Henttonen et al., 2016; Wang et al., 2018). Cheerful employees’ are more inspired, victorious, and connected at a job. This research talks about employees’ FWB. Wang et al. (2018) claim that employees’ optimistic sentiments and personal happiness impel workers to impart their information both tacit and explicit. According to them, when employees are content, they take pleasure in the job and feel optimistic as they can assist co-workers by sharing information with them. As per Chumg et al. (2016), the employees’ happiness increases their “tacit and explicit” attitude relating to KS. According to them, employees who have good associations with their co-workers, which are part of the “eudaemonic” well-being factor, will probably share their knowledge. The employee needs to communicate their economic knowledge and talent with other co-workers to assist others to decide. The knowledge sharing is important as a “moderator.” It is thought that gaining financial literacy will help in talking about financial knowledge, mainly employees’ knowledge and ability to handle other employees to make the right financial selection, enhancing employees’ FWB. Thus, we propose that:

*P6: KS moderates the relationship between financial literacy and FWB.*

## Conceptual Model

**FIGURE 10:**
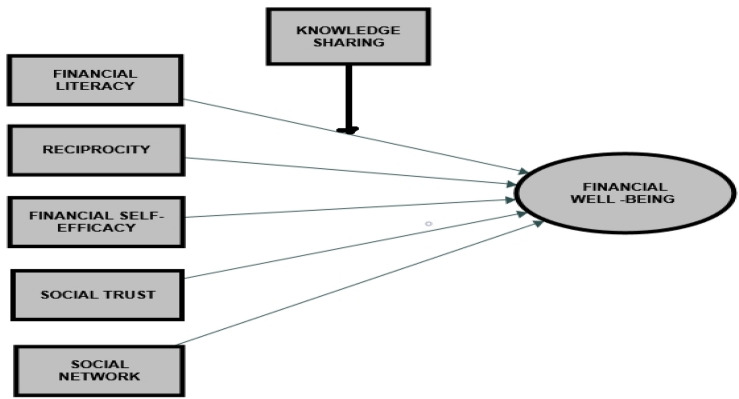
Conceptual model.

A conceptual model is suggested in this literature review-based research to clarify aspects of employees’ FWB. This model is based on three critical social theories. There are two social capital dimensions based on social capital theory, namely, social network (structural social capital) and social trust (relational social capital) with reciprocity that is based on SET. Financial self-efficacy based on “SCT and financial literacy” is the independent variable that promotes employee’s FWB. Many authors have shown the significance of “financial literacy” for promoting “FWB” (Shim et al., 2009; Sabri et al., 2010; Mahendru et al., 2020). In Figure 10, KS may be a moderator connecting employees’ “financial literacy” and “FWB.”

## Conclusion

Financial well-being is being discussed everywhere. FWB is considered significant for academicians, public policy officials, financial managers, and employers. There are low levels of FWB that many employees around the globe experience. FWB contributes significantly to an individual’s happiness. Because of this, there are many workplace FWB programs to address this problem. Thus, the present research is significant in considering the significant factors that can help to improve employees’ FWB in the organization.

Based on SET supposition, organizations’ optimistic and helpful actions toward their employees and/or their representatives help set up the superior quality of associations that confers a duty on an employee to respond positively in a helpful manner. Thus, a manager should appreciate the best probable conversation so that employees are optimistic and the returns are good FWB from their employer. Promoting the employee’s FWB is a type of social accountability, where acts are done to help a larger society. Thus, supporting FWB aids the “corporate social responsibility” (CSR) objectives of an organization to present an excellent organizational picture and trust (Pivato et al., 2008; Vlachos et al., 2019). Many studies have suggested that “image and trust” have an optimistic impact on “profitability.” There is a need for organizations to come out with “financial educators” to work with their employees and offer training as and when needed to educate employees about the difficulties of accruing credit card balances, erroneous savings, and plans to avoid irrational and urged purchase. There is a need to offer official and causal training programs to teach and practice optimistic “credit management behavior and investment management behavior.” Employees should be notified about the likely outcomes of using credit cards, investments, and other financial savings, especially in significant values. Also, employers are required to aid their employees in understanding and learning about indications of irrational and urged purchasing, examine their purchasing strategies, and develop new plans to keep away from difficult purchasing attitudes and redundant expenditures.

The first half of 2020 has impacted both domestic and business due to COVID-19. It is a rigorous investigation of a person’s economic flexibility. Legislators put economic flexibility and FWB at the forefront of the schedule and offer a chance to re-concentrate on the critical aspects of financial education. Also, people/small businesses and their monetary circumstances have faced economic and monetary issues resulting from the health crisis during the COVID-19 pandemic. The extended criticism of financial activity and the long impacts of disrupted monetary connections may affect work, profits, and persons’ economic flexibility. Legislators and organizations have provided momentary advantages to the unemployed and/or easy admission to a person’s money at funded/sponsored price, and provide “credit holidays” where banks are required to postpone the full loan installment payment dates (OECD, 2020). Also, to support an individual’s finances, one suggestion is to provide instant and available guidance on financial education to help people tackle financial anxiety and enhance faith and self-assurance. KS plays a significant role in this as a “moderator” as it is considered that achieving financial education will not assist but communicate the information relating to finances, particularly employees’ know-how information and their knowledge in tackling other employees in coming to a correct economical choice, and the same can augment employees’ FWB. There is a need for financial educators to organize with banks and other monetary institutions to build up social networks that are monetarily friendly to employees to guide them in managing their money properly. There is a requirement to support peer education communication and increase KS of finances where one employee teaches the other about finances and talents and “learn lessons,” contributing to skills in making assets from the peril of exploiting and mistreating their dealings in money. Programs can be incorporated in or connected to employees’ individual monetary training programs. The best option is that financial education must be available with administrators, investigators, practitioners, instructors, and policymakers when they move forward to make their finances and employees’ FWB better (Joo and Grable, 2004). Sustainable vocation entails a long-term viewpoint and concentration on organizational stability. This denotes keeping precious human assets and endorsing the personnel’s abilities. It is vital to expand capability and skill to maintain employability while protecting their money and happiness.

There is a new “employability-based psychological contract,” which mentions no lifetime service in one company or firm (Guest, 2007). Employers cannot be expected to offer them chances to expand their talents and will protect their well-being. Employers require employees to respond to these organization assets by showing dedication and flexibility (Baruch, 2015). It follows that “reciprocity” can arouse modern “organization–employee” associations and have an optimistic influence on vocation sustainability (Florea et al., 2013). The standard of “reciprocity” recommends that employees, when proposed to go after training, like training in finances and other advantages in finances, should restore the benefit by performing for the company or firm’s advantages, e.g., avoid premature retirement. It can be said that a good standard of “reciprocity” can aid in promoting the employee’s FWB and also maintain a good long-term association between employer and employee.

The association between FWB “determinants” and knowledge sharing as a “moderator” will help both industries and employees plan methods to promote FWB between employees. Thus, it is suggested to determine and authenticate the planned hypothetical-based model in the forthcoming study using proposals as a “testable” proposition. Chances to conduct such researches will be there in the developed and developing countries employing diverse methods.

As far as rules and propositions are concerned, financial professionals and policymakers might suggest methods of growing “financial self-efficacy,” “financial literacy,” and significant “social capital dimensions” to aid the population financially, i.e., both vulnerable and invulnerable, and control pressure relating to finances in employment. The present research has two drawbacks. The first is that only the WoS database was employed for bibliometric analysis, and secondly, there is a need for an empirical and systematic review for securing a detailed insight on the subject. Thus, according to the authors, it will be best to conduct a systematic review followed by significant empirical re-examination.

## Author Contributions

AT: conceptualization, methodology, validation, and original draft preparation. AT and VG: visualization and reviewing and editing. VG: supervision. Both authors contributed to the article and approved the submitted version.

## Conflict of Interest

The authors declare that the research was conducted in the absence of any commercial or financial relationships that could be construed as a potential conflict of interest.
